# Plasminogen Activator Inhibitor 1 for Predicting Sepsis Severity and Mortality Outcomes: A Systematic Review and Meta-Analysis

**DOI:** 10.3389/fimmu.2018.01218

**Published:** 2018-06-18

**Authors:** Timothy L. Tipoe, William K. K. Wu, Lilianna Chung, Mengqi Gong, Mei Dong, Tong Liu, Leonardo Roever, Jeffery Ho, Martin C. S. Wong, Matthew T. V. Chan, Gary Tse, Justin C. Y. Wu, Sunny H. Wong

**Affiliations:** ^1^Department of Medicine and Therapeutics, Faculty of Medicine, The Chinese University of Hong Kong, Hong Kong, Hong Kong; ^2^Li Ka Shing Institute of Health Sciences, Faculty of Medicine, The Chinese University of Hong Kong, Hong Kong, Hong Kong; ^3^Department of Anaesthesia and Intensive Care, The Chinese University of Hong Kong, Hong Kong, Hong Kong; ^4^Li Ka Shing Faculty of Medicine, The University of Hong Kong, Hong Kong, Hong Kong; ^5^Tianjin Key Laboratory of Ionic-Molecular Function of Cardiovascular Disease, Department of Cardiology, Tianjin Institute of Cardiology, Second Hospital of Tianjin Medical University, Tianjin, China; ^6^Department of Clinical Research, Federal University of Uberlândia, Uberlândia, Brazil; ^7^JC School of Public Health and Primary Care, Faculty of Medicine, The Chinese University of Hong Kong, Hong Kong, Hong Kong

**Keywords:** plasminogen activator inhibitor-1, sepsis, mortality, meta-analysis, systematic review

## Abstract

**Objectives:**

Plasminogen activator inhibitor-1 (PAI-1), a crucial regulator of fibrinolysis, is increased in sepsis, but its values in predicting disease severity or mortality outcomes have been controversial. Therefore, we conducted a systematic review and meta-analysis of its predictive values in sepsis.

**Methods:**

PubMed and Embase were searched until August 18, 2017 for studies that evaluated the relationships between PAI-1 levels and disease severity or mortality in sepsis.

**Results:**

A total of 112 and 251 entries were retrieved from the databases, of which 18 studies were included in the final meta-analysis. A total of 4,467 patients (36% male, mean age: 62 years, mean follow-up duration: 36 days) were analyzed. PAI-1 levels were significantly higher in non-survivors than survivors [odds ratios (OR): 3.93, 95% confidence interval (CI): 2.31–6.67, *P* < 0.0001] and in patients with severe sepsis than in those less severe sepsis (OR: 3.26, 95% CI: 1.37–7.75, *P* = 0.008).

**Conclusion:**

PAI-1 is a significant predictor of disease severity and all-cause mortality in sepsis. Although the predictive values of PAI-1 reached statistical significance, the clinical utility of PAI-1 in predicting outcomes will require carefully designed prospective trials.

## INTRODUCTION

Sepsis, defined as organ dysfunction caused by a dysregulated host response to infection, is a major cause of morbidity and mortality. Sepsis is often complicated by cardiovascular dysfunction, acute respiratory distress syndrome, and/or multiple organ failure (MOF), which leads to severe sepsis (1). In severe cases, septic shock occurs, which is characterized by profound circulatory abnormalities requiring a vasopressor. Given its clinical importance, extensive investigation has been made on the use of accurate blood biomarkers to predict the severity and mortality in sepsis. Plasminogen activator inhibitor-1 (PAI-1), a crucial regulator of fibrinolysis, has been identified as a potential biomarker. PAI-1 inhibits plasminogen activator, a key enzyme involved in the cleavage of plasminogen to plasmin. This in turn inhibits fibrinolysis, leading to disseminated intravascular coagulation, circulatory hypoperfusion, and organ dysfunction in septic patients.

The major role of PAI-1 in sepsis has subsequently prompted the investigation of PAI-1 as a predictor of disease severity and mortality patients with sepsis. While many studies have explored the role of PAI-1 in this patient subset, few studies have observed differences in PAI-1 levels between sepsis survivors and non-survivors. Moreover, few studies have been conducted to compare PAI-1 levels between patients with septic shock/severe sepsis and those with sepsis alone. This study, therefore, aims to investigate any differences in PAI-1 levels between survivors and non-survivors of sepsis. To achieve this aim, a meta-analysis was performed to systematically evaluate the use of PAI-1 as a biomarker in predicting the severity and mortality of sepsis.

## METHODS

### Search Strategy, Inclusion, and Exclusion Criteria

PubMed and Embase were searched for studies that investigated the relationship between PAI-1 levels at different degrees of the sepsis syndrome, namely, sepsis, severe sepsis, and septic shock. The two databases were also searched for studies that compared PAI-1 levels in non-survivors and survivors of sepsis. The search terms used were “(mortality or hospitalization or severity) AND (plasminogen activator inhibitor-1 OR SERPINE1) AND sepsis,” and the search period was from the beginning of the databases to August 18, 2017, without language restrictions. The inclusion criteria used were a prospective or retrospective cohort study design in humans, and PAI-1 values provided and related to the severity and disease mortality of sepsis patients.

Quality assessment of these studies included in our meta-analysis was performed using the Newcastle–Ottawa Quality Assessment Scale (NOS). The point score system evaluated the categories of study participant selection, comparability of the results, and quality of the outcomes. The following characteristics were assessed: (a) representativeness of the exposed cohort, (b) selection of the non-exposed cohort, (c) ascertainment of exposure, (d) demonstration that outcome of interest was not present at the start of study, (e) comparability of cohorts on the basis of the design or analysis, (f) assessment of outcomes, (g) follow-up period sufficiently long for outcomes to occur, and (h) adequacy of follow-up of cohorts. This scale varied from 0 to 9 stars, which indicated that studies were graded as poor quality if they met <5 criteria, fair if they met 5–7 criteria, and good if they met >8 criteria. The details of the NOS quality assessment are shown in Table S1 in Supplementary Material.

### Data Extraction and Statistical Analysis

Using the lists of generated studies from PubMed and Embase, the articles were reviewed to check for compliance with the mentioned inclusion criteria. Out of all of the studies searched, 26 studies contained data suitable for analysis. The data from these articles were entered into a Microsoft Excel file by two independent reviewers. After further screening, 7 more articles were excluded, leaving a total of 19 suitable articles. For this study, the extracted data elements consist of (i) publication details: surname of first author, publication year; (ii) study design; (iii) follow-up duration; (iv) study endpoint; (v) the characteristics of the population including the sample size, gender, age, and cutoff point for PAI-1 levels where available. The mean PAI-1 values in patients with and without septic shock, as well as between non-survivors and survivors were extracted from each study and subsequently pooled into our meta-analysis. For the relationship between PAI-1 levels and mortality, we extracted and analyzed odds ratios (ORs) and 95% confidence intervals (CI) from each study. Hazard ratios were equated as ORs.

## RESULTS

**Figure 1:**
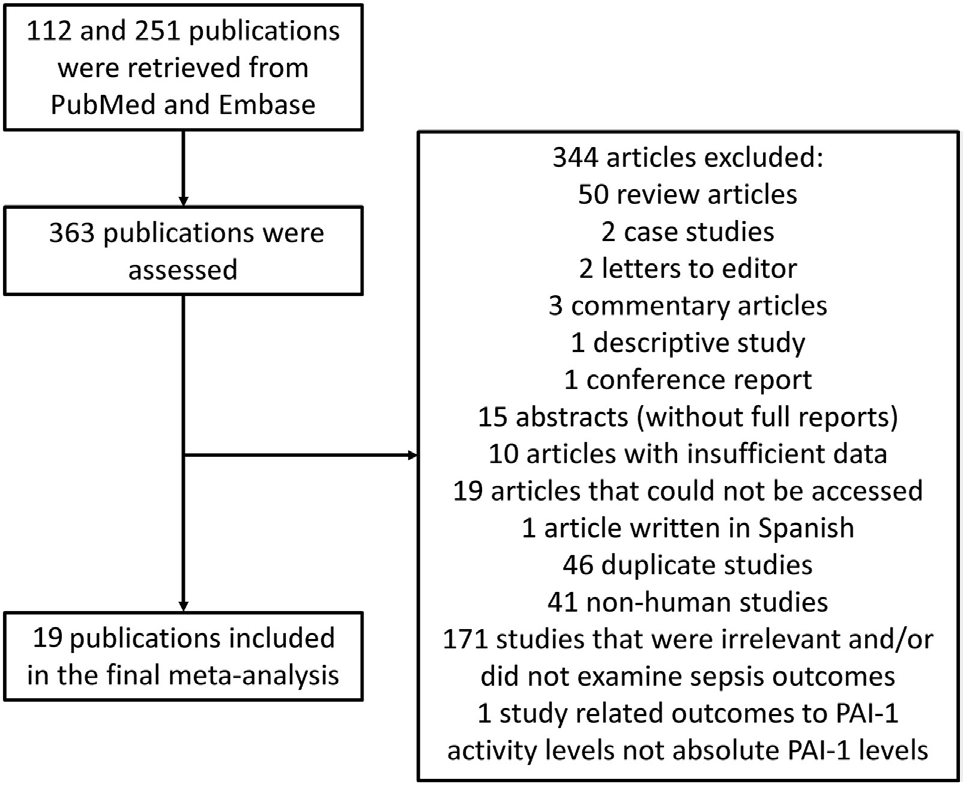
Flow diagram of the study selection process for studies investigating the association between plasminogen activator inhibitor-1 (PAI-1) and outcomes in sepsis.

A flow diagram detailing the search strategy and study selection process is shown in Figure 1. Searches on PubMed and Embase yielded 112 and 251 publications, respectively, of which 19 studies met the inclusion criteria and were included in the final meta-analysis (2–19). The baseline characteristics of these studies are listed in Table 1. All studies apart from one were prospective studies. A total of 4,467 patients (36% male, mean age 62 years; mean follow-up duration of 36 days) were analyzed. In terms of assay type, eight articles used an ELISA kit, two used a sandwich ELISA assay, and two used a bead-based multiplexed immunoassay with the Human Cardiovascular-1 Panel. Other assays used included the latex agglutination test, latex photometric immunoassay, and other chromogenic analyses (Table 1).

**Table 1:** Characteristics of the 19 studies included in the meta-analysis.

First author/year	Population	Assay type	Sample size (*n*)	Age (years)	SD	No. of males	Follow-up (days)	Variables in multivariate model	Reference
Hoshino/2017	Patients with sepsis	STACIA bio-chemical automated clinical testing system (*Mitsubishi Chemical Medience Corp, Tokyo, Japan*)	186	/	/	115	28	Survivors and non-survivors of sepsis	(19)
Koyama/2014	Patients with sepsis in ICU	tPAI latex photometric immuno-assay (*Mitsubishi Chemical Medience, Tokyo, Japan*)	77	69.9	12.9	42	28	Patients with or without septic shock	(14)
Lorente/2014	ICU patients with severe sepsis	ELISA (*American Diagnostica, Inc., Stanford, CT, USA*)	260	59.4	/	170	30	Survivors and non-survivors of sepsis	(15)
Prakash/2015	Sepsis patients with organ failure	Activated human PAI-1 functional assay (*Molecular Innovations, Novi, Mich*)	77	66.2	/	41	3	Survivors of sepsis and septic shock	(18)
Panigada/2015	ICU patients with severe sepsis/septic shock	ELISA kit (*HYPHEN Biomed France*)	40	61	/	24	1	Survivors of sepsis and patients without septic shock	(17)
Lorente/2014	ICU patients with severe sepsis	ELISA (*American Diagnostica, Inc., Stanford, CT, USA*)	295	60.7	/	100	8	Survivors and non-survivors of sepsis	(16)
Seki/2013	Patients with sepsis	PAI-1 latex immune agglutination test (*Mitsubishi Chemical Medicine Corporation, Tokyo, Japan*)	242	71	/	157	28	Survivors and non-survivors of sepsis, and patients with or without septic shock	(13)
Jalkanen/2012	Mechanically ventilated patients in ICU	ELISA (*BioVendor GmbH, Heidelberg, Germany*)	454	64	/	311	90	Survivors and non-survivors of sepsis	(12)
Perés Wingeyer/2011	Caucasian adults with/without sepsis in ICU	Chromogenic analysis (*Diagnostica Stago, France*)	166	/	/	94	/	Survivors and non-survivors of sepsis	(11)
Schuetz/2011	Patients in A&E with an episode of hypertension	Human cardiovascular-1 panel (*Millipore, Billerica, MA, USA*)	116	60	16.7	67	In hospital only	Patients with or without septic shock	(10)
Mauri/2010	Patients with severe sepsis or septic shock	Sandwich ELISA assay	90	61	15	56	90	Survivors and non-survivors of sepsis	(7)
Tsantes/2010	Patients with severe sepsis or septic shock	Automated coagulation analysis (*Behring Coagulation System, Marburg, Germany*) with reagents (*Berichrom PAI; Dade Behring, Milton Keynes, UK*), protocols from the manufacturer	73	62.5	18.1	46	90	Survivors and non-survivors of sepsis, and patients with or without septic shock	(20)
Shapiro/2010	Patients with clinical suspicion of infection	Human cardiovascular-panel (*Millipore, Billerica, MA, USA*)	221	58	19	106	3	Patients with or without septic shock	(8)
Wagenaar/2010	Patients with severe leptospirosis	ELISA (*HYPHEN Biomed France*)	52	45	/	37	5	Survivors and non-survivors of sepsis	(9)
Wiersinga/2008	Patients with culture proven septic melioidosis	ELISA (*TintElize PAI-1, Biopool, Umea, Sweden*)	34	52	/	26	/	Survivors and non-survivors of sepsis	(6)
Zeerleder/2005	Patients with sepsis and septic shock	ELISA (*Milan Analytica, La Roche, Switzerland*)	42	/	/	32	In hospital only	Patients with or without septic, and patients with septic shock	(5)
Prabhakaran/2003	Patients with acute lung injury and sepsis	Sandwich ELISA (*American Diagnostica, Greenwich, CT*)	26	/	/	14	In hospital only	Survivors of sepsis, and patients with septic shock	(3)
Okabayashi/2004	ICU patients	tPAIc test (*Kokusai-Shiyaku*)	1,789	60.9	6.9	48	/	Survivors and non-survivors of sepsis, and patients with or without septic shock	(4)
Raaphorst/2001	Patients with fever	ELISA	300	/	/	153	28	Survivors and non-survivors of sepsis, and patients with or without septic shock	(2)

### PAI-1 for Predicting Disease Severity or Mortality in Sepsis

**Figure 2:**
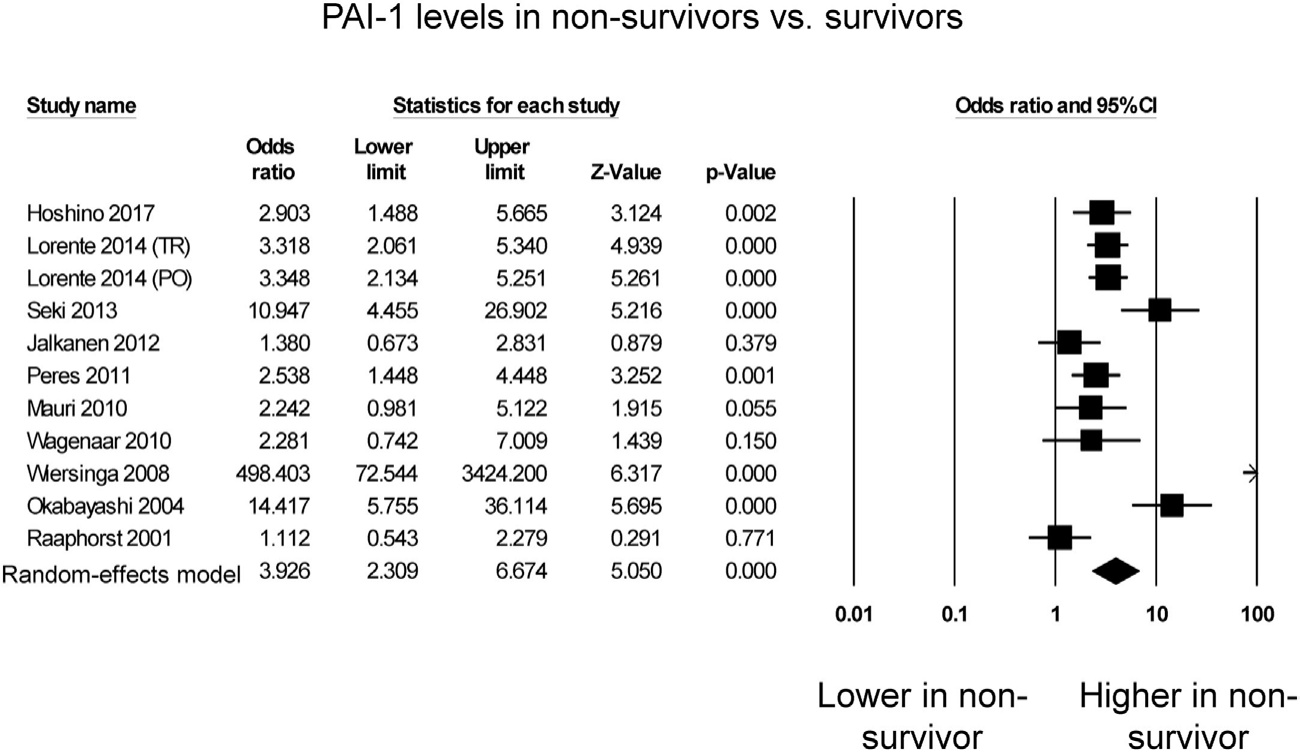
Forest plot comparing plasminogen activator inhibitor-1 (PAI-1) levels between non-survivors and survivors of sepsis.

Eleven studies compared PAI-1 levels between non-survivors and survivors in septic patients. Of these, seven studies reported significantly higher PAI-1 levels in septic patients who died compared with those who survived (Figure 2), whereas four studies reported no significant difference between both groups. Nevertheless, PAI-1 levels were significantly higher in non-survivors than survivors (OR: 3.93, 95% CI: 2.31–6.67, *P* < 0.0001). *I*^2^ took a value of 83%, indicating presence of substantial heterogeneity. Sensitivity analysis excluding one study at a time did not significantly affect the pooled estimate (Figure S1 in Supplementary Material). A funnel plot of SE against the logarithm of odds ratio is shown in Figure S2 in Supplementary Material. Begg and Mazumdar rank correlation suggested no significant publication bias (Kendal’s Tau value 0.2, *P* = 0.39). Egger’s test demonstrated no significant asymmetry (intercept 3.5, *t*-value 1.7; *P* = 0.12).

**Figure 3:**
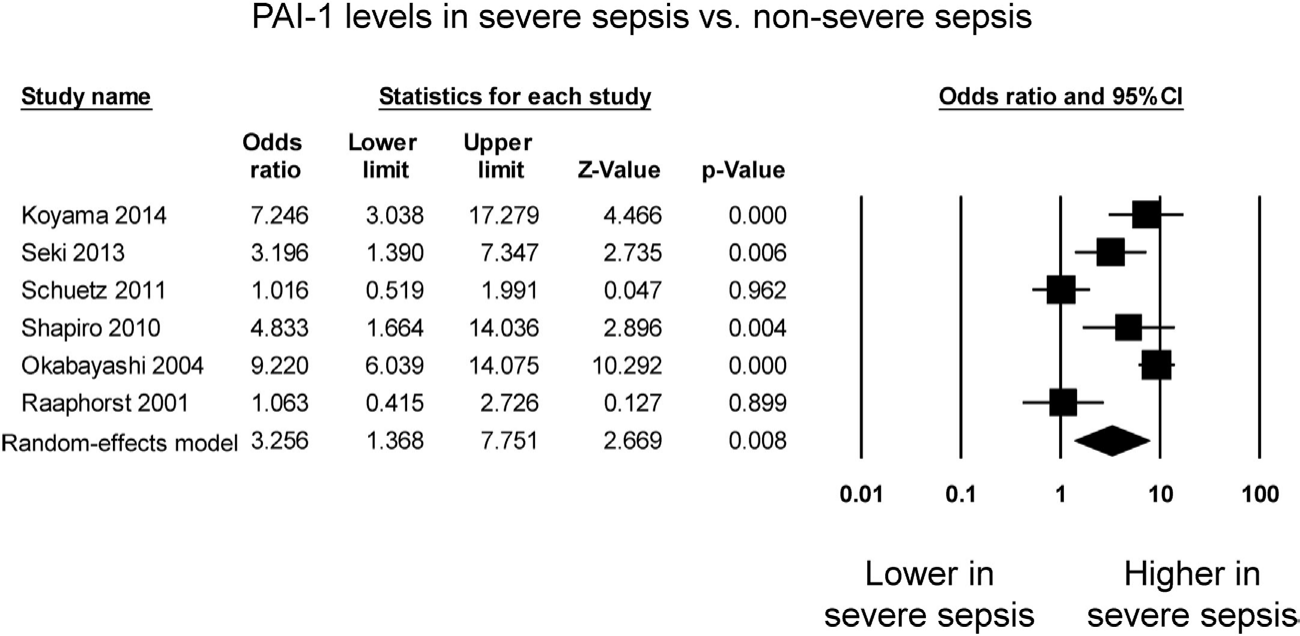
Forest plot comparing plasminogen activator inhibitor-1 (PAI-1) levels between patients with severe sepsis and those with non-severe sepsis.

Six studies compared PAI-1 levels between patients with severe sepsis and patients with less severe sepsis. Four studies reported significant higher levels in the case of severe sepsis whereas the remaining two studies reported no significant difference between the groups (Figure 3). PAI-1 levels were significantly higher in patients with severe sepsis than in those less severe sepsis (OR: 3.26, 95% CI: 1.37–7.75, *P* = 0.008). *I*^2^ took a value of 88%, indicating that substantial heterogeneity was present. Sensitivity analysis excluding one study at a time did not significantly affect the pooled estimate (Figure S3 in Supplementary Material). A funnel plot of SE against the logarithm of odds ratio is shown in Figure S4 in Supplementary Material. Begg and Mazumdar rank correlation suggested no significant publication bias (Kendal’s Tau value −0.07, *P* = 0.85). Egger’s test demonstrated no significant asymmetry (intercept −4.2, *t*-value 1.2; *P* = 0.28).

## DISCUSSION

The main findings of this systematic review and meta-analysis are that higher level of PAI-1 are observed in patients with severe sepsis compared with less severe sepsis, and in non-survivors compared with survivors.

Sepsis is a potentially life-threatening condition and is often complicated by MOF involving excessive activation of coagulation (21). Regardless of the inciting pathogen (21, 22), endothelial activation and inflammation are found to be key in the initiation and continuation of host response, which primarily determine clinical outcomes within septic patients (22). Endothelial expression of tissue factor induced by a myriad of pro-inflammatory cytokines leads to subsequent systemic pro-coagulant response and activation of the anti-fibrinolytic pathway (23, 24). With the coagulation cascade inadequately contained by natural anti-coagulant reaction, the shift toward pro-coagulant state results in excessive thrombin generation, microvascular fibrin deposition, and consumption of clotting factors, a process termed disseminated intravascular coagulation, contributing to significant morbidity and mortality secondary to associated MOF and coagulopathy (23, 25). In severe sepsis, fibrinolysis and coagulation inhibitors are depleted, supported by a decrease in major coagulation inhibitors (e.g., PC and anti-thrombin) and plasminogen, along with an increase in PAI-1, our marker of interest, as shown in previous studies (21, 26–27).

Plasminogen activator inhibitor-1, as the primary inhibitor of both tissue-type and urokinase-type plasminogen activators (t-TPA and u-TPA), inhibits fibrinolysis and is associated with various vascular complications. Functionally active at its native conformation upon its release from endothelial cells, PAI-1 exerts inhibitory effects toward u-TPA and t-TPA with a functional half-life of 12 h at 37°C in normal conditions (28). In multiple clinical studies, the increase of PAI-1 was shown to correlate with sepsis severity and mortality (5, 29–31).

However, few studies have demonstrated significant differences of PAI-1 within different patient groups. The findings of this meta-analysis provide further support to the role of PAI-1 to guide clinical management. For example, those patients with initially high PAI-1 levels may be offered a more proactive management, including early ICU admission, initiation of fluid resuscitation, and inotropic support. Its diagnostic and prognostic value in combination with other plasma biomarkers needs to be elucidated in future studies (32–34).

### Limitations

We must also acknowledge several limitations in the study. Substantial heterogeneity was observed in three meta-analyses with *I*^2^ larger than 75%. A potential source of this heterogeneity could be due to the differences in study designs, collection times, and quantification assays for PAI-1. Funnel plots also showed significant asymmetry to suggest publication bias. Finally, some studies used univariate analysis, meaning that some data may have some confounding factors. Moreover, it is worth noting that not only is PAI-1 synthesized in a wide range of tissue but also takes different inter-convertible conformations, of which its stability and functional activity widely varies (35–37). Moreover, the influence of PAI-1 on pathophysiology of sepsis may differ across populations, affected by genetic and environmental factors (38–40). Hence, another limitation of using PAI-1 in sepsis lies in the difficulty in interpreting the biomarker level with its biochemical properties taken into account. Further studies on the effects of genetic polymorphism and environmental conditions on the biochemical profile of the biomarker can be conducted to establish the role of PAI-1 in different clinical conditions, particularly sepsis in which deranged homeostasis is evident.

## CONCLUSION

A high level of PAI-1 distinguishes non-survivor from survivors for sepsis, consistent with its statistically significant correlation with all-cause mortality. Moreover, higher PAI-1 levels were observed in patients with severe sepsis than those with sepsis. Future prospective studies or trials should focus on the predictive power of combining PAI-1 and existing classic clinical severity scores such as SOFA and APACHE II for guiding clinical management of sepsis.

## DATA AVAILABILITY STATEMENT

The raw data supporting the conclusions of this manuscript will be made available by the authors, without undue reservation, upon request to the corresponding authors.

## AUTHOR CONTRIBUTIONS

TT and WW acquired, analyzed, and interpreted the data. LC, MG, MD, TL, LR, JH, MW, and MC provided important technical and intellectual contents. GT, JW, and SW conceived, designed, and oversaw this study. TT, WW, and GT drafted the manuscript, and all authors revised and approved the final manuscript.

## Conflict of Interest Statement

The authors declare that the research was conducted in the absence of any commercial or financial relationships that could be construed as a potential conflict of interest.
