# Supplementary material for: Plasminogen Activator Inhibitor 1 for Predicting Sepsis Severity and Mortality Outcomes: A Systematic Review and Meta-Analysis
Source: Front Immunol. 2018 Jun 18;9:1218. doi: 10.3389/fimmu.2018.01218 (PMC6015919; doi:10.3389/fimmu.2018.01218)
Supplement: Supplementary file 1 [file Data_Sheet_1.PDF]

Supplementary Figures

PAI-1 levels for non-survivors vs. survivors: sensitivity analysis

Study name	Statistics with study removed				
	Point	Lower limit	Upper limit	Z-Value	p-Value
Hoshino 2017	4.126	2.284	7.453	4.698	0.000
Lorente 2014 (TR)	4.124	2.221	7.659	4.486	0.000
Lorente 2014 (PO)	4.131	2.212	7.713	4.452	0.000
Seki 2013	3.524	2.058	6.036	4.589	0.000
Jalkanen 2012	4.394	2.509	7.695	5.178	0.000
Peres 2011	4.215	2.310	7.688	4.690	0.000
Mauri 2010	4.197	2.358	7.469	4.877	0.000
Wagenaar 2010	4.137	2.350	7.282	4.922	0.000
Wiersinga 2008	3.068	2.034	4.630	5.343	0.000
Okabayashi 2004	3.407	2.025	5.731	4.619	0.000
Raaphorst 2001	4.457	2.588	7.675	5.388	0.000
Random-effects model	3.926	2.309	6.674	5.050	0.000

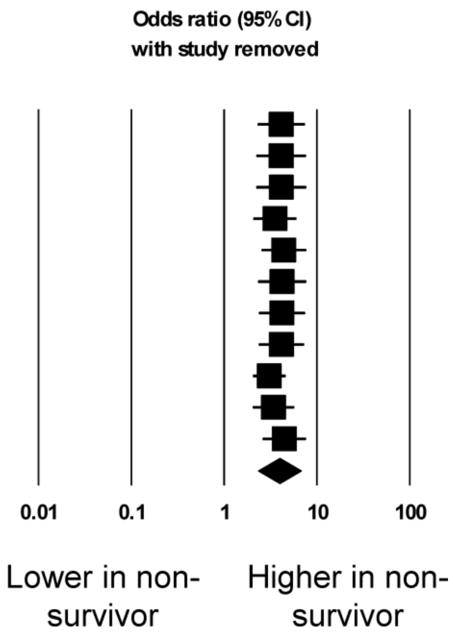

Supplementary Figure 1. Sensitivity analysis for the comparisons between PAI-1 levels in non-survivors and survivors of sepsis.

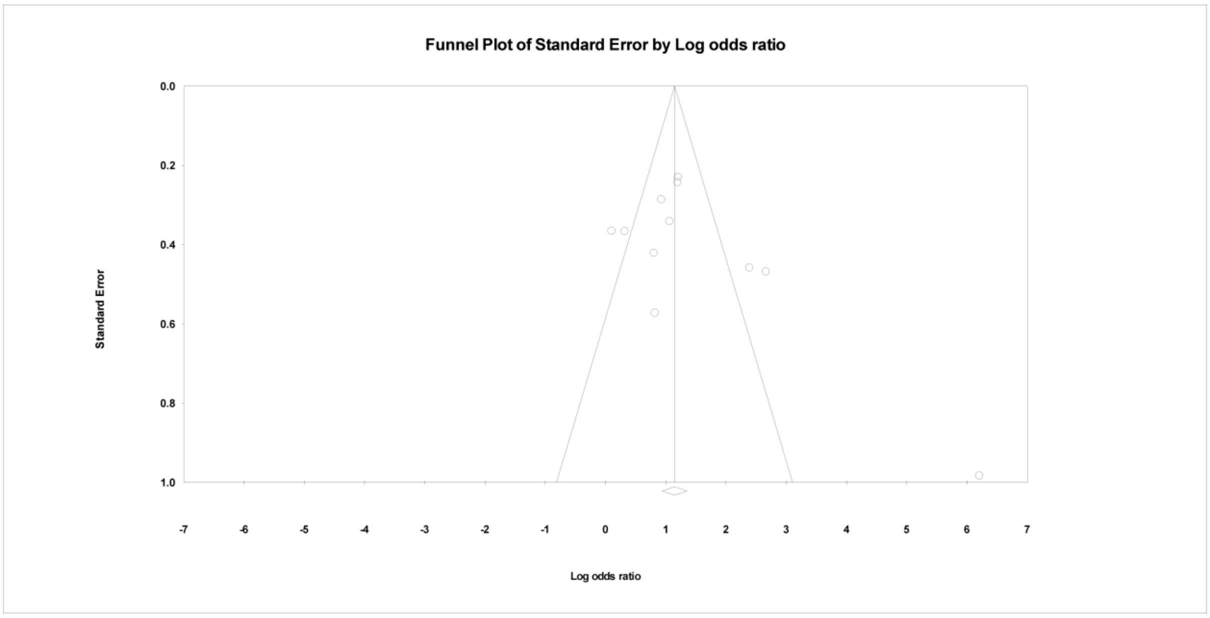

Supplementary Figure 2. Funnel plot of standard error against the logarithm of odds ratio for the comparisons between PAI-1 levels in non-survivors and survivors of sepsis.

PAI-1 levels in severe sepsis vs. non-severe sepsis: sensitivity analysis

Study name	Statistics with study removed				
	Point	Lower limit	Upper limit	Z-Value	p-Value
Koyama 2014	2.778	1.002	7.703	1.964	0.050
Seki 2013	3.260	1.149	9.248	2.221	0.026
Schuetz 2011	4.237	1.961	9.155	3.674	0.000
Shapiro 2010	3.027	1.109	8.267	2.161	0.031
Okabayashi 2004	2.545	1.139	5.686	2.277	0.023
Raaphorst 2001	4.024	1.629	9.942	3.017	0.003
Random-effects model	3.256	1.368	7.751	2.669	0.008

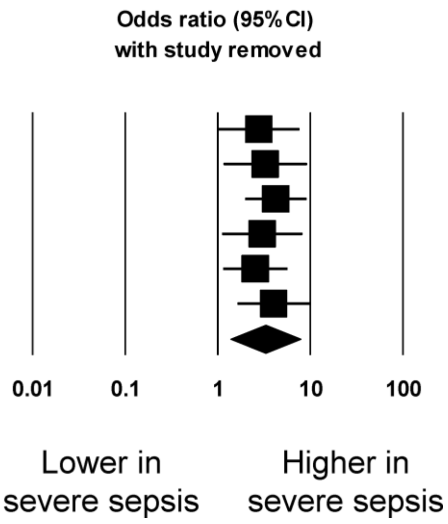

Supplementary Figure 3. Sensitivity analysis for the comparisons between PAI-1 levels in patients with severe sepsis and in those with non-severe sepsis.

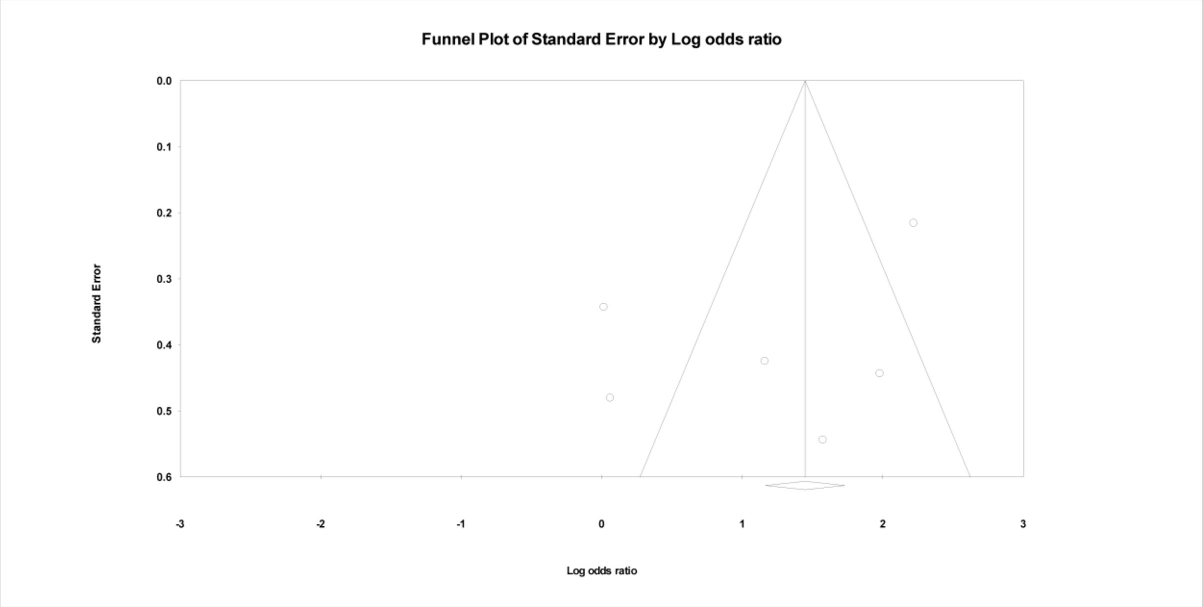

Supplementary Figure 4. Funnel plot of standard error against the logarithm of odds ratio for the comparisons between PAI-1 levels in patients with severe sepsis and in those with non-severe sepsis.
