# Supplementary material for: Plasminogen Activator Inhibitor 1 for Predicting Sepsis Severity and Mortality Outcomes: A Systematic Review and Meta-Analysis
Source: Front Immunol. 2018 Jun 18;9:1218. doi: 10.3389/fimmu.2018.01218 (PMC6015919; doi:10.3389/fimmu.2018.01218)
Supplement: Supplementary file 2 [file Table_1.docx]

NOS risk of bias scale for included cohort studies

|  |  | Selection |  |  |  |  | Outcome |  |  |
| --- | --- | --- | --- | --- | --- | --- | --- | --- | --- |
| First author / Year | Representativeness of the exposed cohort | Selection of the non-exposed cohort | Ascertainment of exposure | Outcome of interest not present at start of study | Comparability | Assessment of outcome | Adequacy of duration of follow-up | Adequacy of completeness of follow-up | Total score  (0-9) |
| Hoshino 2017 | 1 | 0 | 1 | 0 | 0 | 1 | 1 | 1 | 5 |
| Jalkanen 2012 | 1 | 0 | 1 | 1 | 1 (age) | 1 | 1 | 1 | 7 |
| Koyama 2014 | 1 | 0 | 1 | 1 | 1 (age) | 1 | 1 | 1 | 7 |
| Lorente 2014 (PO) | 1 | 0 | 1 | 1 | 0 | 1 | 1 | 1 | 6 |
| Lorente 2014 (TR) | 1 | 0 | 1 | 1 | 0 | 1 | 1 | 1 | 6 |
| Mauri 2010 | 1 | 0 | 1 | 1 | 2 (age, co-morbidities) | 1 | 1 | 1 | 8 |
| Okabayashi 2004 | 1 | 0 | 1 | 1 | 0 | 1 | 1 | 1 | 6 |
| Panigada 2015 | 1 | 0 | 1 | 1 | 0 | 1 | 1 | 1 | 6 |
| Perés Wingeyer 2011 | 1 | 1 | 1 | 1 | 1 (co-morbidities) | 1 | 1 | 1 | 8 |
| Prabhakaran 2003 | 1 | 0 | 1 | 1 | 1 (age) | 1 | 1 | 1 | 7 |
| Prakash 2015 | 1 | 0 | 1 | 1 | 1 (age) | 1 | 1 | 1 | 7 |
| Rssphirst 2001 | 1 | 0 | 1 | 1 | 0 | 1 | 1 | 1 | 6 |
| Schuetz 2011 | 1 | 0 | 1 | 1 | 0 | 1 | 1 | 1 | 6 |
| Seki 2013 | 1 | 0 | 1 | 1 | 0 | 1 | 1 | 1 | 6 |
| Shapiro 2010 | 1 | 0 | 1 | 1 | 0 | 1 | 1 | 1 | 6 |
| Tsantes 2010 | 1 | 0 | 1 | 1 | 0 | 1 | 1 | 1 | 6 |
| Wagenaar 2010 | 1 | 0 | 1 | 1 | 0 | 1 | 1 | 1 | 6 |
| Wiersinga 2008 | 1 | 0 | 1 | 1 | 0 | 1 | 1 | 1 | 6 |
| Zeerleder 2005 | 1 | 0 | 1 | 1 | 0 | 1 | 1 | 1 | 6 |
